# Studying Kidney Diseases Using Organoid Models

**DOI:** 10.3389/fcell.2022.845401

**Published:** 2022-03-03

**Authors:** Meng Liu, Angelysia Cardilla, Joanne Ngeow, Ximing Gong, Yun Xia

**Affiliations:** ^1^ Lee Kong Chian School of Medicine, Nanyang Technological University Singapore, Singapore, Singapore; ^2^ Cancer Genetics Service, National Cancer Centre Singapore, Singapore, Singapore

**Keywords:** kidney organoid, kidney disease, iPSC, disease modelling, differentiation, tubuloid, tumoroid, PKD

## Abstract

The prevalence of chronic kidney disease (CKD) is rapidly increasing over the last few decades, owing to the global increase in diabetes, and cardiovascular diseases. Dialysis greatly compromises the life quality of patients, while demand for transplantable kidney cannot be met, underscoring the need to develop novel therapeutic approaches to stop or reverse CKD progression. Our understanding of kidney disease is primarily derived from studies using animal models and cell culture. While cross-species differences made it challenging to fully translate findings from animal models into clinical practice, primary patient cells quickly lose the original phenotypes during *in vitro* culture. Over the last decade, remarkable achievements have been made for generating 3-dimensional (3D) miniature organs (organoids) by exposing stem cells to culture conditions that mimic the signaling cues required for the development of a particular organ or tissue. 3D kidney organoids have been successfully generated from different types of source cells, including human pluripotent stem cells (hPSCs), adult/fetal renal tissues, and kidney cancer biopsy. Alongside gene editing tools, hPSC-derived kidney organoids are being harnessed to model genetic kidney diseases. In comparison, adult kidney-derived tubuloids and kidney cancer-derived tumoroids are still in their infancy. Herein, we first summarize the currently available kidney organoid models. Next, we discuss recent advances in kidney disease modelling using organoid models. Finally, we consider the major challenges that have hindered the application of kidney organoids in disease modelling and drug evaluation and propose prospective solutions.

## Introduction

The global prevalence of Chronic Kidney Disease (CKD) and End Stage Renal Disease (ESRD) is increasing with an alarming rate (Go et al., 2004; Bikbov et al., 2020). The clinical presentation of CKD and ESRD is often associated with cardiovascular diseases, diabetes, and hypertension (Gansevoort et al., 2013; Matsushita et al., 2015; Webster et al., 2017). Currently, hemodialysis and transplantation remain to be the primary treatment options for ESRD. Dialysis substantially reduces patients’ life quality, while the availability of transplantable kidney is consistently insufficient (Tonelli et al., 2011). These limitations strongly suggest that there is an urgency to develop new therapeutic approaches to fight the global burden of kidney diseases.

A better understanding of the mechanistic underpinning of CKD will help develop novel treatments and preventive methods. The underlying causes of CKD can be broadly classified into genetic and non-genetic. Genetic kidney diseases are caused by mutations of single or multiple genes, being those germline inherited or somatically acquired. Examples of genetic kidney diseases include polycystic kidney disease (PKD), glomerular nephropathy, and renal cancer (Hildebrandt, 2010). Non-genetic kidney diseases can lead to acute kidney injury (AKI), which may be caused by infection, toxic chemicals, or systemic vascular complication such as diabetes and hypertension, although these could be associated with genetic factors as well (Thomas et al., 2015; Makris and Spanou, 2016).

Traditionally, animal models, and monolayer cell culture have been employed to understand kidney development and disease. Undoubtedly, these models have a profound impact on the way we approach disease modelling and drug discovery. Nevertheless, knowledge derived from traditional model systems cannot always be extrapolated to human due to interspecies differences. Organoids are a cluster of cells that self-organize into three-dimensional (3D) structures and could recapitulate critical features of the cognate organ. Organoids can be derived from human pluripotent stem cells (hPSCs) and adult/fetal tissues. The last decade has witnessed the explosion of disease modelling studies employing organoids as the model system, including kidney organoids. Despite numerous limitations of kidney organoids, including the fetal-like state and existence of off-target cells, they are by far the most physiologically relevant model of human kidney. Here, we first revisit the “conventional” models for kidney diseases, followed by discussion of the most prevailing approaches for generating different types of kidney organoids. Then, we consider the current utility and limitation of kidney organoids for disease modelling, as well as contemplate future prospects in generating kidney organoids of higher physiological relevance for faithfully recapitulating kidney diseases.

## “Conventional” Models for Studying Kidney Diseases

Mouse models have been extensively used to recapitulate kidney diseases due to the evolutionarily conserved developmental program, involving reciprocal interaction between metanephric mesenchyme (MM) and ureteric bud (UB), as well as the similarity in organ architecture and physiological function (Kim & Dressler, 2005; Taguchi et al., 2014; Takasato & Little, 2015; McMahon, 2016). Indeed, mutations in genes that are crucial for mouse kidney development are associated with congenital anomalies of the kidney and urinary tract (CAKUT) syndromes in human (Hwang et al., 2014; Nicolaou et al., 2015). The recent development of CRISPR/Cas9 genome editing tools greatly facilitated the investigation of human kidney diseases with complex genetic traits (Li et al., 2013; Sander & Joung, 2014).

Polycystic kidney disease (PKD) represents one of the most common monogenic kidney diseases, constituting approximately 3% of CKD cases. PKD can be mainly categorised into autosomal dominant (ADPKD) and autosomal recessive (ARPKD) (Wilson, 2004), typically induced by germline mutation of *PKD1* or *PKD2*, and *PKHD1*, respectively (Qian et al., 1996; Pei et al., 1999). Because homozygous germline deletion of *Pkd1* or *Pkd2* in mice lead to embryonic lethality, conditional/kidney-specific knockout, and hypomorphic models are more suitable to investigate ADPKD pathogenesis (Herron et al., 2002; Leeuwen et al., 2004; Piontek et al., 2004; Piontek et al., 2007; Yu et al., 2007; Takakura et al., 2008). PCK rats harbouring *Pck* mutation progressively develop cysts in distal tubule and collecting duct, hence are often used for ARPKD studies (Lager et al., 2001). Although genetic PKD mouse models recapitulate key pathological features of PKD, they cannot emulate many complex mechanisms such as the “second-hit” action in ADPKD and the highly variable disease severity caused by different mutations (Happé and Peters, 2014). Moreover, mTOR inhibitors sirolimus and everolimus failed the clinical trial despite the beneficial effects observed in PKD mouse models, highlighting interspecies differences in disease mechanism (Serra et al., 2010; Walz et al., 2010). Whole exome sequencing has identified a list of novel genes associated with PKD, such as *GANAB* and *DNAJB11* in ADPKD and *DZIP1L* in ARPKD (Lu et al., 2017), requiring new models for interrogating the roles of these new genes in PKD pathogenesis.

Glomerular nephropathy is characterized by disruption of glomerular filtration, such as focal segmental glomerulosclerosis (FSGS) and IgA nephropathy (Meyrier, 2005; Roberts, 2014). FSGS mouse models have been established by introducing orthologous genetic aberrations identified in patients (Kaplan et al., 2000; Mele et al., 2011; Plageman et al., 2011). While genetic mouse models (knockouts of *Actn4* or *Myo1e*) that recapitulate the secondary forms of FSGS are available, primary FSGS models with unknown cause are lacking (Henderson et al., 2008; Krendel et al., 2009). Spontaneous ddY mouse and CD89 transgenic mouse have been widely used for modelling IgA nephropathy. However, they couldn’t mirror complex real-life scenarios in different patients (Launay et al., 2000; Coppo et al., 2002; Moura et al., 2008). Spontaneous lupus mouse strain NZB/NZWF1 (BW) develops glomerulonephritis and vasculitis, thus has been widely used for lupus study (Eilat et al., 1976; Satoh et al., 1995; Neubert et al., 2008; Mukundan et al., 2009; Li et al., 2011). Nevertheless, differences between mouse and human in immune system activation and response to challenge, in both the innate and adaptive arms, suggest us to exercise extra caution when we translate paradigms in mouse to human.

Both genetic and non-genetic mouse models are available for studying diabetic nephropathy (DN). While type I diabetes is most commonly induced by streptozotocin-mediated pancreatic *β*-cells ablation (Rossini et al., 1978; Toniolo et al., 1980; Leiter, 1982), type II diabetes mouse model is usually developed by genetic modification of leptin receptor (Maffei et al., 1995; Lee et al., 1996). Unfortunately, the correlation between genetic background and phenotype severity in different mouse strains remains an obstacle for DN studies (Brosius et al., 2009; Brosius and Alpers, 2013; Betz and Conway, 2016). Acute kidney injury (AKI) can be induced by ischemia-reperfusion injury (IRI), drug toxicity or sepsis (Andres et al., 1962; Zwacka et al., 1998; Piliponsky et al., 2008). The most commonly used IRI mouse models are unstable, due to variable surgery proficiency. It is important to establish reproducible AKI models that are independent of human errors.

Renal cancer studies have employed either mouse models with genetic modification of oncogenes and/or tumour suppressors (Kapitsinou & Haase, 2008; Wang et al., 2014; Gu et al., 2017; Harlander et al., 2017; Nargund et al., 2017), or xenograft models with tumour biopsies derived from patients (Prochazka et al., 1992; Lee & Motzer, 2017). Nevertheless, these models are far from being able to recapitulate human renal tumour microenvironment.

In addition to mouse models, conventional 2-dimensional (2D) monolayer cell culture has contributed substantially to our understanding of kidney diseases. Patient-derived primary kidney cells provide the opportunity to study donor-specific phenotypes. However, limited expansion capability and complicated tissue isolation process have prompted the generation of immortalized cell lines, wherein cells are genetically modified to acquire indefinite proliferation capability, such as HK2 human proximal tubular epithelial cells (Ryan et al., 1994). To study podocytes, a major breakthrough came with the establishment of immortalized temperature-sensitive podocytes where cell growth and differentiation could be regulated under specific condition (Mundel et al., 1997; Saleem et al., 2002). Despite its simplicity, accessibility and low cost, monolayer cell culture has numerous limitations. Among these, deprivation of 3D tissue architecture prohibited the recapitulation of disease phenotypes that involve cell-cell or cell-extracellular matrix (ECM) interaction. To this point, collagen I matrix embedding has enabled MDCK cells to form 3D polarized tubular structure with lumen formation, mimicking renal epithelial cysts in PKD (Mangoo-Karim et al., 1989; Yamaguchi et al., 1995; Boletta et al., 2000; O’Brien et al., 2002). Although these conventional models of kidney diseases will continue to contribute to our knowledge, the limitations of these models have raised the urgent need for developing new models that could faithfully emulate human kidney diseases, being those genetic or non-genetic.

## Establishment of Kidney Organoids 

**FIGURE 1:**
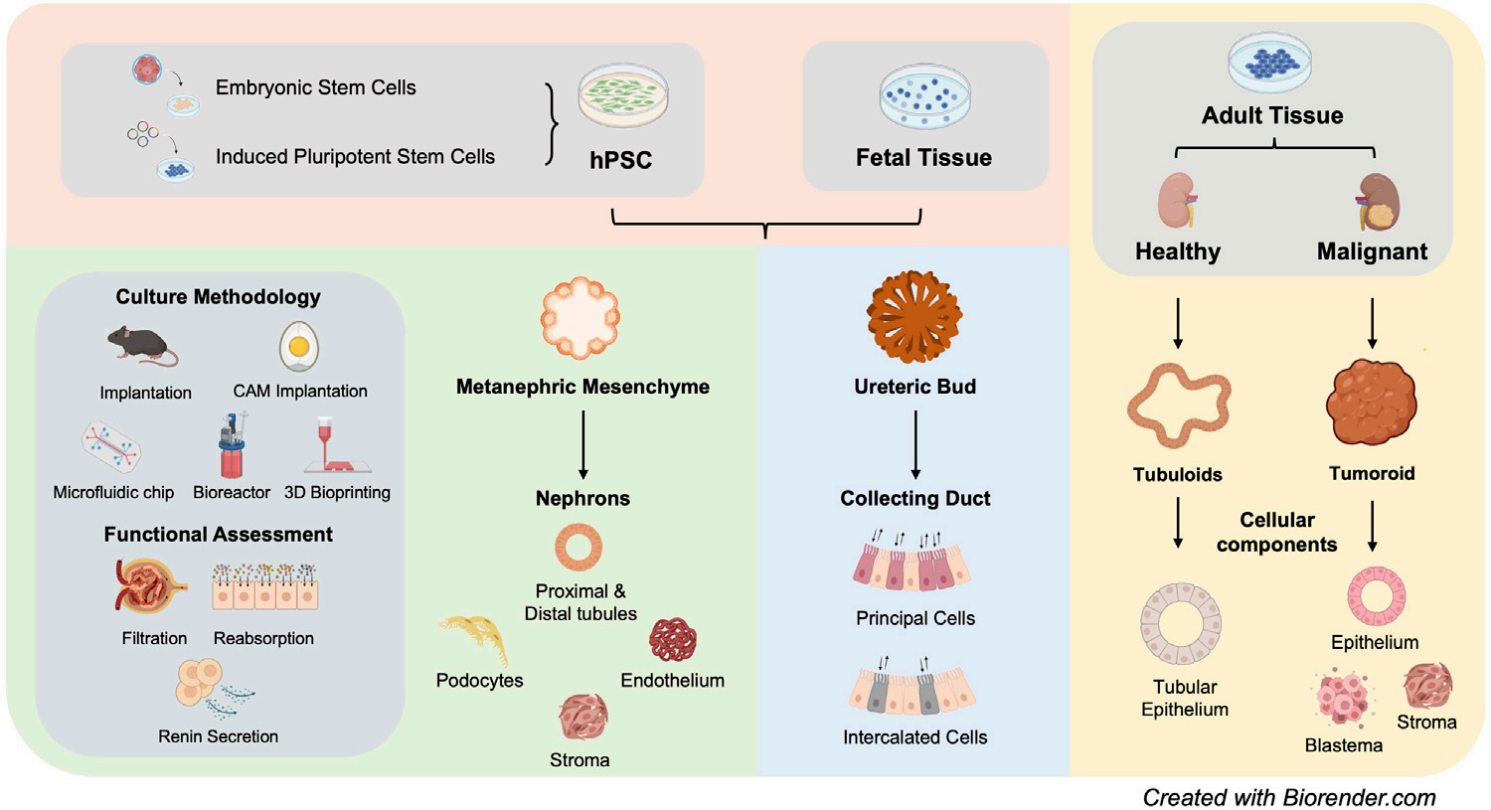
Summary of current kideny organoid models (hPSC: human pluripotent stem cells).

The last decade has borne witness to a large body of studies that aim to differentiate hPSCs into kidney organoids that present both the cellular repertoire and 3D structure of human kidney. Earlier study using mouse embryonic stem cells (ESCs) provided valuable insights into the specific biochemical signals required for renal lineage commitment (Kim & Dressler, 2005). In 2013, three studies demonstrated successful differentiation of hPSCs into MM or UB lineages that are capable of self-organizing into 3D tubular structures upon either aggregation with mouse embryonic kidney cells or co-culture with mouse embryonic spinal cord (Mae et al., 2013; Xia et al., 2013; Taguchi et al., 2014). Moving forward, *Takasato et al* reported simultaneous derivation of both MM and UB from hPSCs, followed by self-organization into 3D kidney tubular structures in the absence of embryonic mouse tissues (Takasato et al., 2014). At the end of 2015, two seminal studies demonstrated for the first time that hPSCs can be efficiently differentiated into self-assembled 3D kidney organoids (Morizane et al., 2015; Takasato et al., 2015). Figure 1

Substantial structural and functional characterization of hPSC-derived kidney organoids have been performed. Overall, these organoids are comprised of segmentally patterned nephron-like structures, stromal cells and endothelial cells, showing high congruence with human fetal kidney. Nevertheless, these kidney organoids presented rudimentary function, such as tubular reabsorption represented by proximal tubule epithelium-mediated dextran uptake (Freedman et al., 2015; Takasato et al., 2015; Przepiorski et al., 2018; Low et al., 2019) and secretion of functional renin (Shankar et al., 2021). Within kidney organoids, glomerular podocytes adopt the structural conformation reminiscent of glomerulus, facilitating selective isolation, and enrichment of glomerulus-like structures for studying nephrotic syndrome (Hale et al., 2018; Yoshimura et al., 2019). Due to the highly complex cell composition, kidney organoids, alongside single-cell RNA-sequencing (scRNAseq), enabled characterization of inter-cellular cross-talk and disease relevance, pointing out new directions for future exploration (Wu et al., 2018; Low et al., 2019; Ungricht et al., 2021).

Kidney is a filtration organ, the functionality of which is indispensable of a patterned vascular network. Although endothelial cells could be generated alongside nephron epithelium using these differentiation protocols (Freedman et al., 2015; Takasato et al., 2015), they are under-represented. Various approaches have been developed to vascularize hPSC-derived kidney organoids. VEGF-A, being those exogenously administered into differentiation culture (Czerniecki et al., 2018) or those autologously generated by podocytes (Low et al., 2019), greatly facilitated kidney organoid vascularization. Despite the existence of a rich vascular network within kidney organoids, most glomeruli remained avascular (Low et al., 2019). Engraftment of kidney organoids into immune-compromised mouse (Bantounas et al., 2018; van den Berg et al., 2018; Low et al., 2019) or chick chorioallantoic membrane (CAM) (Garreta et al., 2019) facilitated glomerular vascularization. The grafted kidney organoids not only got anastomosed with the host circulation system, but also established putative glomerular filtration barrier. Most importantly, the grafted kidney organoids were capable of handling systemically injected dextran in a size-selective manner, indicating functional maturation. However, engraftment of kidney organoids into model organisms largely limited their downstream utility. To this point, *Homan et al* developed a microfluidic chip culture that employed fluid flow to enhance vascularization and maturation of kidney organoids, circumventing the necessity of a host circulation system (Homan et al., 2019). Despite successful vascular invasion into glomerulus, kidney organoids did not display a similar level of vascularization in comparison with the transplanted organoids.

There has been a long-standing debate about whether UB lineage exists within hPSC-derived kidney organoids, despite the existence of GATA3^+^ tubule population. UB is originated from anterior intermediate mesoderm, different from MM which is originated from posterior intermediate mesoderm. In 2017, a landmark study demonstrated successful generation of UB organoids with a single collecting duct tree, forming properly patterned renal macro-anatomy upon aggregation with mouse PSC-derived MM and embryonic mouse kidney stromal progenitor cells (Taguchi and Nishinakamura, 2017). Recently, a number of UB differentiation protocols have been developed with improvements considering long-term culture maintenance, stable formation of expandable branching epithelium, as well as maturation into collecting duct-like structures (Mae et al., 2020; Uchimura et al., 2020; Zeng et al., 2021). Recent single cell transcriptomic analyses of both mouse and human embryonic kidneys revealed that many UB lineage markers, including *GATA3* and *AQP2*, are expressed in distal connecting tubules that are supposed to be descendants of MM (Combes et al., 2019a). Based on a widely-used protocol for kidney organoid generation, a recent study developed an alternative approach to induce ureteric epithelium identity, via harnessing the cellular plasticity of distal nephrons that were derived from MM kidney organoids (Howden et al., 2021). Whether the observed cellular plasticity of distal nephron reproduces normal development or homeostasis of human kidney warrants further investigation.

Apart from hPSC-derived kidney organoids, both fetal and adult renal tissues can give rise to organoids, when provided with a suitable culture condition. Mouse and human embryonic kidney derived UB and MM cells can be captured for long-term *in vitro* culture within a synthetic niche. MM cells of different origins, including mouse/human embryonic kidneys and hPSCs, could be kept in non-differentiated progenitor state while remain competent to differentiate into segmentally patterned nephron structures (Brown et al., 2015; Li et al., 2016). Likewise, synthetic niche has been recently developed for UB cells from mouse embryonic origin or hPSCs (Yuri et al., 2017; Zeng et al., 2021). In 2019, Clevers team developed the first protocol to generate kidney organoids, termed tubuloids, from adult human kidney tubular epithelial cells and urine-derived tubular epithelial cells. Tubuloids, grown in semi-solid ECM, are comprised of a mixed population of kidney epithelium, adopting distinctive apical-basal polarity (Schutgens et al., 2019). Alternatively, tubular epithelial cells could be generated from fibroblasts via transcription factor-directed reprogramming alongside the action of defined growth factors (Kaminski et al., 2016). Both approaches allow long-term maintenance and stable expansion of healthy adult kidney epithelium *ex vivo,* providing opportunities to study kidney tissue regeneration*.*

Tumoroids have been generated from many different types of malignant tissues, such as colorectal tumour (Fujii et al., 2016), liver tumour (Broutier et al., 2017), and ovarian tumour (Kopper et al., 2019). Recently, the establishment of tumoroids from renal tumour biopsy has been reported by several groups, demonstrating long-term propagation of tumoroids with defined culture cocktail (Grassi et al., 2019; Schutgens et al., 2019; Fendler et al., 2020). Kidney tumoroids recapitulate the heterogeneity of the parental tumour tissue, displaying tri-phasic histology of epithelial, stromal, and blastema components. Different culture condition results in distinctive *in vitro* characteristics of tumoroids, such as expansion capacity and tissue morphology (Fendler et al., 2020). The technical feasibility for tumoroid generation has enabled biobanking of kidney tumoroids (Calandrini et al., 2020).

The availability of various kidney organoid models has opened a new avenue for modelling human kidney diseases, including genetic diseases, infection and nephrotoxicity, within a 3D tissue microenvironment (Figure 1).

## Organoid Models of Kidney Diseases

### Polycystic Kidney Disease

Human PSCs are highly amenable for genetic manipulation, making it feasible to introduce genetic aberrations that are associated with genetic kidney diseases. Among these, PKD has been most frequently studied using hPSC-derived kidney organoids. In ADPKD patients, cysts are primarily located in the proximal tubules, while ARPKD patients have more collecting duct cysts. Nevertheless, fetal stage cysts of ARPKD patients are often observed in proximal tubules. Towards the late stage of PKD, cysts are observed along the entire length of nephrons (Bergmann et al., 2018). *Freedman et al* introduced truncating mutations of *PKD1* or *PKD2* into hPSCs and differentiated them into kidney organoids (Freedman et al., 2015). Although spontaneous cyst formation was observed in PKD knockout organoids after long term culture (∼Day 58), cystogenesis was not efficient under adhesion culture condition (∼6%) presumably due to different tissue microenvironment between *in vivo* and *in vitro* systems. To improve cystogenesis, Freedman group applied suspension culture, leading to a 10-fold increase in the efficiency of cystogenesis (Cruz et al., 2017). The cystic kidney organoids presented cellular phenotypes of ADPKD, including fluid accumulation and proliferation of cyst-lining epithelial cells. This study also demonstrated a previously unregistered role of adhesive microenvironment in restraining cyst dilation during the early stage of PKD.

The question remains as to what extent patient iPSC- or gene-edited PSC-derived cystic kidney organoids recapitulate PKD pathology. It is quite common that stress paradigm is required for patient iPSC derivatives to present disease phenotypes that typically manifest during adulthood. Decades of studies using animal models and monolayer cell culture have clearly demonstrated aberrant intracellular levels of cAMP and Ca^2+^ in PKD (Torres and Harris, 2014). Hence, forskolin, a potent activator of adenylyl cyclase (AC), is frequently used to induce cyst formation in kidney organoids that are derived from patient iPSCs or gene-edited hPSCs. *Shimizu et al* generated kidney organoids from ADPKD patient iPSCs, and cysts were predominantly observed in proximal tubules following exposure to forskolin (Shimizu et al., 2020). On the contrary, *Cruz et al* observed no striking difference between PKD and non-PKD PSC-derived kidney organoids upon cAMP induction using forskolin or 8-Br-cAMP (Cruz et al., 2017). *Kuraoka et al* generated UB organoids from both ADPKD patient iPSCs and gene-edited *PKD1* mutant iPSCs. Cyst formation was observed in UB stalk region after forskolin treatment, recapitulating the initial stage of ADPKD cystogenesis (Koptides and Deltas, 2000; Kuraoka et al., 2020)*.*

Comparing with ADPKD, ARPKD has a lower incidence and typically manifests at fetal or neonatal stage. *Low et al* generated kidney organoids from ARPKD patient iPSCs and gene-corrected isogenic iPSCs. Cyst dilation was specifically observed in patient iPSC-derived kidney organoids, first in the proximal region and then extended to the distal region after forskolin or 8-Br-cAMP stimulation, recapitulating gestational cyst formation of ARPKD (Nakanishi et al., 2000; Gunay-Aygun et al., 2006; Woollard et al., 2007; Low et al., 2019). In a recent study, *Howden et al* obtained ureteric epithelium culture via a detour from *PKHD1* ^null^ iPSC-derived MM organoids and observed spontaneous cyst formation under ureteric stalk culture condition, mimicking ureteric epithelium-originated cyst in ARPKD (Howden et al., 2021). Collecting duct cyst formation is usually attributed to vasopressin-mediated activation of AVPR2, which preferentially couples with Gs to activate AC, leading to cAMP production (Boertien et al., 2013). *Kuraoka et al* observed cyst formation in UB organoids after vasopressin treatment, due to expression of *AVPR1A* instead of *AVPR2,* further implying the immaturity of UB organoids (Boertien et al., 2013; Kuraoka et al., 2020). The proliferation phenotype of cystic epithelium is also associated with activation of mitogen-activated protein kinase/extracellular regulated kinase (MAPK/ERK) signalling. To this point, *Shimizu et al* employed epidermal growth factor (EGF) to activate the MAPK/ERK pathway to initiate cyst formation by boosting cell proliferation (Shimizu et al., 2020). However, only a slight increase of organoid size was detected instead of obvious cyst formation, indicating a minor contribution of MAPK/ERK signalling to cyst initiation.

PKD organoid models offer great prospects to evaluate the therapeutic effects of candidate drugs. Cystic fibrosis transmembrane conductance regulator (CFTR) mediates fluid accumulation during cystogenesis (Davidow et al., 1996). CFTR inhibitor successfully blocked cyst formation in ADPKD and ARPKD patient iPSC-derived kidney organoids (Low et al., 2019; Shimizu et al., 2020). Thapsigargin, which inhibits sarco/endoplasmic reticulum Ca^2+^ ATPase, also repressed forskolin-induced cyst formation in ARPKD organoids (Low et al., 2019). In another study, everolimus, which showed encouraging results in mouse model, albeit failed to retard disease progression in ADPKD patients, significantly suppressed forskolin-induced cyst formation in ADPKD organoids (Shimizu et al., 2020). In addition to validating compounds with known effects on cystogenesis, cystic kidney organoids also enabled screening of compounds that have unknown effects on cyst formation. Blebbistatin, a non-muscle myosin II inhibitor, can significantly induce cyst formation in PKD organoids (Czerniecki et al., 2018). These studies highlight the capability of kidney organoids in recapitulating critical PKD machinery, enabling exploration of novel PKD pathways and screening for new drugs.

### Other Genetic Kidney Diseases

Genetic lesions that lead to defective glomerular structure, such as defects in glomerular basement membrane (GBM) and loss of slit diaphragms (SD), can cause proteinuria and/or haematuria (Boute et al., 2000; Daehn and Duffield, 2021). Understanding of glomerular nephropathy is hampered by complex 3D structure of glomerulus and limited proliferation capacity of podocytes. hPSC-derived kidney organoids provide the possibility to overcome these limitations. In a proof-of-concept study, *PODXL* ^*−/−*^ hPSCs were generated by gene editing for studying human glomerular development. The results showed Podocalyxin plays critical roles in microvillus formation and cell spacing in hPSC-derived podocytes (Doyonnas et al., 2001; Kim et al., 2017). Genetic mouse model study further corroborated these results (Kim et al., 2017). *NPHS1* encodes NEPHRIN protein, which is a major component of SD. Mutations in *NPHS1* were initially identified in patients with Finnish-type congenital nephrotic syndrome (CNS) (Patrakka et al., 2000). *Tanigawa et al* used iPSCs from patients with *NPHS1* mutation to study SD formation. Podocytes derived from *NPHS1* mutant patient iPSCs exhibited reduced cell surface localization of NEPHRIN. Upon implantation beneath mouse renal capsule, NPHS1 was also absent at cell junction, despite the formation of well-organized foot processes. Genetic correction of *NPSH1* mutation successfully restored SD formation (Tanigawa et al., 2018). In a separate study, *Hale et al* isolated and enriched glomerular-like structures from hPSC-derived kidney organoids to study CNS, and observed hypertrophied podocytes in *NPHS1* mutant patient-derived organoids (Hale et al., 2018).

Mucin 1 kidney disease (MKD) is a toxic proteinopathy caused by *MUC1* frameshift (MUC1-fs) mutation (Kirby et al., 2013). *Dvela-Levitt et al* showed that MUC1-fs accumulated in TMED9 vesicles between *cis*-Golgi and ER in MKD patient iPSC-derived kidney organoids. The effect of BRD4780 on MUC1-fs protein levels was tested in MKD patient iPSC-derived kidney organoids. Similar with the observation in mouse disease model, BRD4780 managed to clear the mutant protein from intracellular compartments in patient-derived organoids. (Dvela-Levitt et al., 2019).

Cystinosis is a rare lysosomal-storage disease associated with accumulation of cystine in renal proximal tubule, mainly caused by mutations in cystine transporter *CYSTINOSIN* (*CTNS*). *CTNS* ^−/-^ patient iPSC-derived kidney organoids exhibited enlarged lysosome, elevated cystine accumulation, increased apoptosis, and perturbed basal autophagy flux. Dual treatment of cysteamine/everolimus is more effective in slowing down disease progression than single treatment, providing potential therapeutic strategy (Hollywood et al., 2020).

### Renal Cancer

Recent development and characterization of renal cancer-derived tumoroids demonstrated successful preservation of critical genetic and phenotypic features of parental tumour tissues. Clear cell renal cell carcinoma-derived tumoroids contained epithelial and mesenchymal cells with renal cancer specific marker expression such as HIF1α. The tumoroids remain propagative after xenotransplantation (Grassi et al., 2019). *Calandrini et al* established a biobank from various childhood kidney cancers including Wilms tumours, renal cell carcinomas (RCC), malignant rhabdoid tumours of the kidney (MRTK), etc. These tumoroids displayed tri-phasic histology of epithelial, stromal and blastema components. In addition, MRTK tumoroids represent the first cancer organoid that can sustain long-term *in vitro* expansion of tumours of non-epithelial origin (Calandrini et al., 2020). Research using renal cancer-derived tumoroids is still in its infancy, requiring further improvement of the methodology for downstream applications.

### Non-Genetic Kidney Diseases

Human PSC-derived kidney organoids have been less often used for modelling non-genetic diseases, possibly due to immaturity, the lack of blood perfusion and fluid flow, and the absence of immune components. Nonetheless, several studies successfully employed kidney organoids for investigating pathogen-renal interaction. BK virus infection is a common cause of kidney transplant failure (Hirsch et al., 2005). *Schutgens et al* infected adult renal epithelium-derived tubuloids with BK virus. The infected tubuloids presented enlarged nuclei and detectable DNA fragment, reminiscent of BK nephropathy. Furthermore, a clinically used DNA polymerase inhibitor cidofovir significantly decreased BK copy number in infected tubuloids (Schutgens et al., 2019).

Severe acute respiratory syndrome coronavirus 2 (SARS-CoV-2) is responsible for the ongoing global pandemic. Patients with hypertension and diabetes have a higher risk of severe symptoms (Fang et al., 2020), though the underlying mechanism remains unclear. Angiotensin converting enzyme 2 (ACE2), the principle target of SARS-CoV-2, is also expressed in kidney (Vaduganathan et al., 2020). Human recombinant ACE2 effectively reduced the number of infected cells in kidney organoids (Monteil et al., 2020). Human kidney organoids have also been employed to test a novel soluble human ACE2 protein, 1–618-ABD. The results suggested potent neutralization of SARS-CoV-2 by soluble ACE2 1-618-ABD (Wysocki et al., 2021). Furthermore, a recent study revealed SARS-CoV-2 infection drives fibrosis in kidney organoids, corroborating the association between COVID-19 and kidney fibrosis in some patients (Jansen et al., 2021).

Drug-induced nephrotoxicity represents a significant contributor to acute kidney injury (AKI) and CKD. Kidney organoids can respond to different drugs or environmental stresses by expressing tissue specific injury markers. While gentamycin and cisplatin induced kidney injury marker 1 (KIM1) expression and tubular epithelium apoptosis in hPSCs-derived kidney organoid (Freedman et al., 2015; Morizane et al., 2015; Takasato et al., 2015), adriamycin treatment caused podocyte damage and loss (Kumar et al., 2019; Lawlor et al., 2021). These studies revealed previously unappreciated features of kidney organoids, further expanding their utility for modelling a wider spectrum of kidney diseases, as well as for drug screening and toxicological studies.

## Limitations of Kidney Organoids for Disease Modelling

**FIGURE 2:**
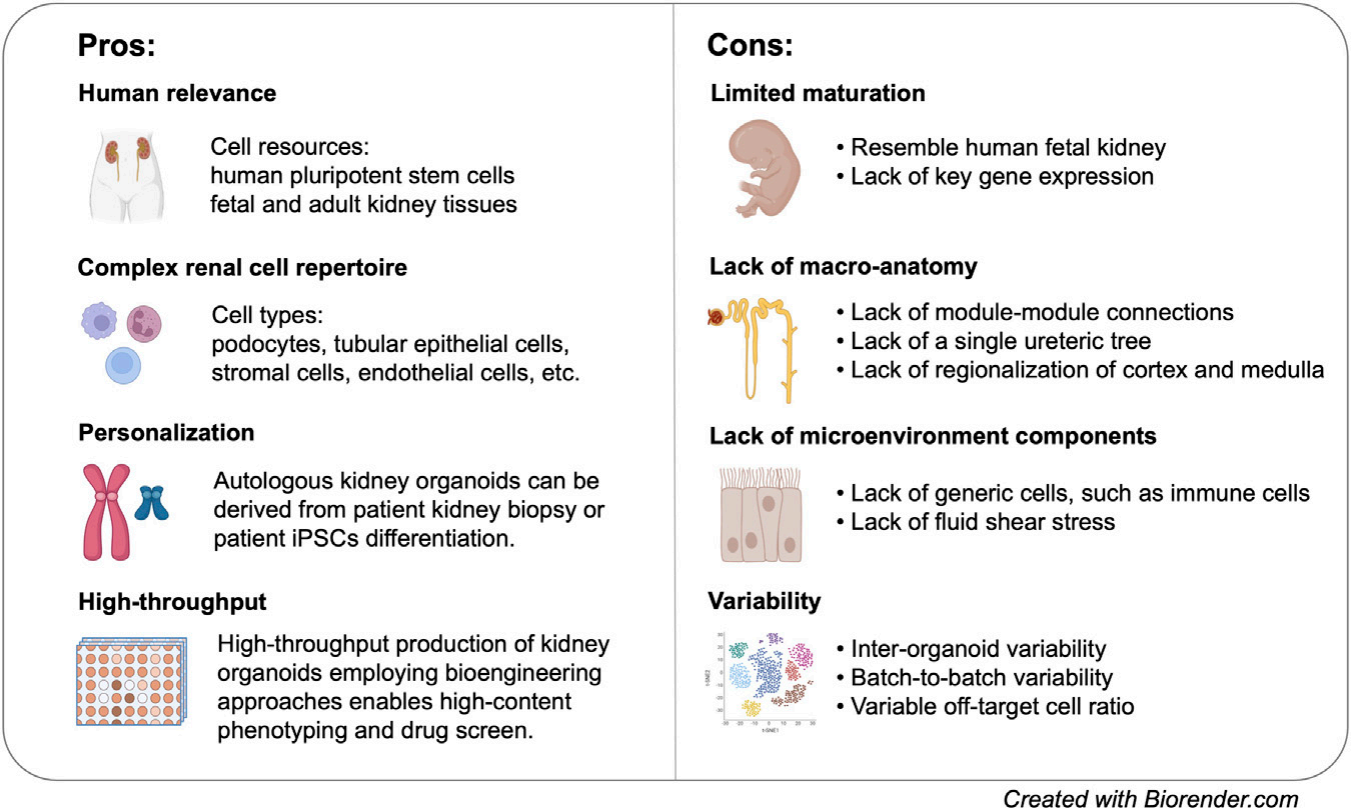
Pros and cons of kideny organoid models for disease modelling.

Global transcriptomic analysis of hPSC-derived kidney organoids indicated that these organoids resemble first/second trimester human fetal kidney, making it an ideal model to study kidney development (Takasato et al., 2015; Garreta et al., 2019). Nevertheless, *in vitro* differentiation of kidney organoids does not necessarily follow the same trajectory as *in vivo* development. As a simple matter of fact, glomerulus-like structures from hPSCs can be formed in 3 weeks of time, while the first layers of human metanephric glomeruli are detected around 7 weeks after fertilization. The recently discovered cellular plasticity of distal nephron segments may or may not reflect *in vivo* lineage relationship. scRNA-seq further revealed the absence of key functional proteins in specific cells associated with kidney-related disorders (Park et al., 2018; Wu et al., 2018). Tolvaptan, the only FDA-approved drug for inhibiting cyst dilation, targets AVPR2 in the collecting duct (Torres et al., 2017). However, modulation of vasopressin receptor in kidney organoid is mostly through *AVPR1A* due to the lack of *AVPR2* expression in current kidney organoid models (Freedman et al., 2015; Kuraoka et al., 2020). Likewise, scRNA-seq revealed minimal detection of *OCT2* transporter in proximal tubule that mediates the uptake of numerous chemical compounds and drugs, such as metformin and cisplatin (Wu et al., 2018; Combes et al., 2019b). Although cisplatin induces proximal tubule damage in hPSC-derived kidney organoids, there is no clear evidence whether the uptake was mediated via the “cognate” transporter (Freedman et al., 2015; Morizane et al., 2015).(Figure 2).

Single cell analysis greatly facilitated the revelation of cellular diversity of kidney organoids. Indeed, kidney organoids contain podocytes, proximal tubule, loop of Henle (LoH), distal tubule, and stromal populations (Czerniecki et al., 2018; Wu et al., 2018; Combes et al., 2019b). Although nephron segment-specific cells are present in MM kidney organoid, there is lack of specification within each of the segments, such as proximal tubular segmentation or the establishment of descending, and ascending LoH (Kanai et al., 1994; Cristofori et al., 2007). Unbiased single cell analysis showed that organoid variability can be attributed to the presence of off-target cells and variations in temporal maturation (Czerniecki et al., 2018; Wu et al., 2018; Combes et al., 2019b). The relative abundance of renal and non-renal cells may also be highly variable (Wu et al., 2018; Phipson et al., 2019). When modelling genetic diseases, inter-organoid variability and batch-to-batch variability may lead to confounding observation when we compare disease phenotypes between patient-derived and isogenic control organoids. In one of the studies, day 18 patient derived organoid with *IFT140* mutation was unexpectedly most similar to day 25 control organoid (Phipson et al., 2019).

Most importantly, generic cell populations are severely under-represented in hPSC-derived kidney organoids, including different types of vascular endothelial cells, renal stroma (England et al., 2020), immune components, etc. Many adult-onset kidney diseases are intimately associated with these “non-renal” cells. For example, kidney fibrosis is characterized by phenotypic change in renal stroma and an excessive production of ECM, ultimately leading to renal failure. In a recent study, transcriptomic analysis of day 35 wild-type kidney organoids suggested that kidney fibrosis is on the way (Ungricht et al., 2021). This characteristic requires us to exercise extra caution in evaluating the fibrotic status using organoid models. The absence of immune components within organoid represents a major limitation for modelling infectious diseases as it precludes the possibility to study renal-immune interaction and autoimmune kidney diseases. Although many studies demonstrated that kidney organoids are “infectable”, the presented phenotypes may be over-simplified comparing with *in vivo* scenarios.

Another major limitation of kidney organoids is the lack of close-to-native macro-anatomy. The 3D arrangement of current kidney organoids makes it impossible to access renal function that requires higher-order organ architecture, such as renal filtration, tubular reabsorption, and urine concentration. Most UB organoids, except those derived from mouse ESCs using protocol developed by Nishinakamura group (Taguchi and Nishinakamura, 2017), do not harbour a single trunk ureteric tree. Furthermore, none of the UB protocols has realized bifurcation of more than two branching events (Taguchi and Nishinakamura, 2017; Uchimura et al., 2020; Zeng et al., 2021). Very recently, Nishinakamura group succeeded in generating high-order kidney organoids via differentiating mouse ESCs separately into nephron progenitors, UB progenitors, and stromal progenitors followed by aggregating all progenitors together (Tanigawa et al., 2022). Although it remains a distant goal to generate kidney organoids with regionalized cortex and medulla, the current achievements may bring us towards the ultimate goal in a few years.

Among all the kidney organoids we have discussed here, adult renal tissue-derived organoids have not been extensively used for modelling kidney diseases, possibly due to the technical challenges in generating tubuloids and tumoroids. Renal cancer-derived tumoroids offer a new means for evaluating prospective drug effects in a patient-specific manner. However, out of the few drugs tested, not all drugs show similar reaction in tumoroids as previously exhibited in primary tumours (Grassi et al., 2019). Moreover, different mutations and previous exposure to chemo-drugs could confer distinctive dose-response curve in tumoroids (Calandrini et al., 2020).

In spite of all these limitations, kidney organoids represent a unique model system that enables us to interrogate human-specific kidney disease phenotypes with a resolution that has never been achieved (Figure 2). Nevertheless, the explosion of organoid biology does not take away the value of conventional models. On the contrary, the availability of different model systems allows researchers to leverage on results obtained from different models, leading to even more comprehensive interpretation of kidney diseases.

## Perspective

The establishment of kidney organoids have provided unprecedented opportunities for modelling various types of human kidney diseases with complex pathological phenotypes. Human PSC-derived kidney organoids have shown remarkable advantages in presenting phenotypes involving complex tissue architecture while retaining patient genetic composition, ushering a new era of personalized medicine. To address the limitations of hPSC-derived kidney organoids, multiple bioengineering approaches are being developed and incorporated into organoid culture. Microfluidic device has successfully facilitated vascularization and maturation of *in vitro* kidney organoid culture (Homan et al., 2019). Furthermore, microfluidic device has also enabled the functional interaction between nephron epithelial and vascular cells, which is required for the realization of proximal tubular reabsorption (Lin et al., 2019) and glomerular filtration (Musah et al., 2017). To circumvent inter-organoid variability, as well as to scale up organoid production, several high-throughput culture methods are available, including 3D extrusion bioprinting (Lawlor et al., 2021), microwell culture (Czerniecki et al., 2018), and suspension bioreactor culture (Przepiorski et al., 2018; Kumar et al., 2019).

Comparing with hPSC-derived kidney organoids, adult renal tissue-derived organoids are expected to show greater potential in modelling kidney diseases that manifest during adulthood. While patient-derived tubuloids facilitate the investigation of genetic diseases and infectious diseases (Schutgens et al., 2019), healthy adult-derived tubuloids may provide a novel model system for studying renal tubule regeneration. In comparison with animal models or cancer cell lines, kidney cancer-derived tumoroids represent a great alternative for studying tumor heterogeneity and progression, as well as for patient-specific drug validation. Although much remains to be done for efficient and consistent derivation of tumoroids from kidney cancer biopsies, patient-specific tumoroids offer exciting opportunities to look into the interaction between tumor cells and autologous immune cells, enabling immune-oncology investigation within the tumor microenvironment and personalized immunotherapy testing (Neal et al., 2018).

During the last ten exciting years, the development of novel organoid models has substantially expanded our capability to investigate human kidney development and diseases within a 3D tissue microenvironment *in vitro*. Alongside animal models and monolayer cell culture models, kidney organoids will undoubtedly advance our understanding of kidney diseases and facilitate the development of novel therapeutics.
